# Humoral immune responses to COVID-19 vaccination in people living with HIV receiving suppressive antiretroviral therapy

**DOI:** 10.1038/s41541-022-00452-6

**Published:** 2022-02-28

**Authors:** Zabrina L. Brumme, Francis Mwimanzi, Hope R. Lapointe, Peter K. Cheung, Yurou Sang, Maggie C. Duncan, Fatima Yaseen, Olga Agafitei, Siobhan Ennis, Kurtis Ng, Simran Basra, Li Yi Lim, Rebecca Kalikawe, Sarah Speckmaier, Nadia Moran-Garcia, Landon Young, Hesham Ali, Bruce Ganase, Gisele Umviligihozo, F. Harrison Omondi, Kieran Atkinson, Hanwei Sudderuddin, Junine Toy, Paul Sereda, Laura Burns, Cecilia T. Costiniuk, Curtis Cooper, Aslam H. Anis, Victor Leung, Daniel Holmes, Mari L. DeMarco, Janet Simons, Malcolm Hedgcock, Marc G. Romney, Rolando Barrios, Silvia Guillemi, Chanson J. Brumme, Ralph Pantophlet, Julio S. G. Montaner, Masahiro Niikura, Marianne Harris, Mark Hull, Mark A. Brockman

**Affiliations:** 1 Faculty of Health Sciences, Simon Fraser University, Burnaby, Canada; 2 British Columbia Centre for Excellence in HIV/AIDS, Vancouver, Canada; 3 Department of Molecular Biology and Biochemistry, Simon Fraser University, Burnaby, Canada; 4 Department of Chemistry, Simon Fraser University, Burnaby, Canada; 5 Division of Medical Microbiology and Virology, St. Paul’s Hospital, Vancouver, Canada; 6 John Ruedy Clinic, St, Paul’s Hospital, Vancouver, Canada; 7 AIDS Research Program, St. Paul’s Hospital, Vancouver, Canada; 8 Department of Medicine, University of British Columbia, Vancouver, Canada; 9 Department of Pathology and Laboratory Medicine, Providence Health Care, Vancouver, Canada; 10 Division of Infectious Diseases and Chronic Viral Illness Service, McGill University Health Centre and Research Institute of the McGill University Health Centre, Montreal, QC Canada; 11 Department of Medicine, University of Ottawa, Ottawa, Canada; 12 Ottawa Hospital Research Institute, Ottawa, Canada; 13 School of Population and Public Health, University of British Columbia, Vancouver, Canada; 14 CIHR Canadian HIV Trials Network, University of British Columbia, Vancouver, Canada; 15 Centre for Health Evaluation and Outcome Sciences, Vancouver, Canada; 16 Department of Pathology and Laboratory Medicine, University of British Columbia, Vancouver, Canada; 17 Spectrum Health, Vancouver, Canada; 18 Department of Family Practice, Faculty of Medicine, University of British Columbia, Vancouver, Canada

**Keywords:** HIV infections, Outcomes research, Vaccines

## Abstract

Humoral responses to COVID-19 vaccines in people living with HIV (PLWH) remain incompletely characterized. We measured circulating antibodies against the SARS-CoV-2 spike protein receptor-binding domain (RBD), ACE2 displacement and viral neutralization activities one month following the first and second COVID-19 vaccine doses, and again 3 months following the second dose, in 100 adult PLWH and 152 controls. All PLWH were receiving suppressive antiretroviral therapy, with median CD4+ T-cell counts of 710 (IQR 525–935) cells/mm^3^, though nadir CD4+ T-cell counts ranged as low as <10 cells/mm^3^. After adjustment for sociodemographic, health and vaccine-related variables, HIV infection was associated with lower anti-RBD antibody concentrations and ACE2 displacement activity after one vaccine dose. Following two doses however, HIV was not significantly associated with the magnitude of any humoral response after multivariable adjustment. Rather, older age, a higher burden of chronic health conditions, and dual ChAdOx1 vaccination were associated with lower responses after two vaccine doses. No significant correlation was observed between recent or nadir CD4+ T-cell counts and responses to two vaccine doses in PLWH. These results indicate that PLWH with well-controlled viral loads and CD4+ T-cell counts in a healthy range generally mount strong initial humoral responses to dual COVID-19 vaccination. Factors including age, co-morbidities, vaccine brand, response durability and the rise of new SARS-CoV-2 variants will influence when PLWH will benefit from additional doses. Further studies of PLWH who are not receiving antiretroviral treatment or who have low CD4+ T-cell counts are needed, as are longer-term assessments of response durability.

## Introduction

COVID-19 vaccination is expected to benefit people living with HIV (PLWH)^1^, since they may be at increased risk for severe COVID-19 as a result of immunosuppression, higher rates of multi-morbidity or social determinants of health^2–5^. Our understanding of immune responses to COVID-19 immunization in PLWH however remains incomplete, in part because relatively few PLWH were included in the clinical trials for the COVID-19 vaccines that have now been widely administered in Canada and Europe (~196 for the BNT162b2 mRNA vaccine^6,7^, 176 for the mRNA-1273 mRNA vaccine^8^ and 54 and 103 PLWH respectively in the UK and South Africa for the ChAdOx1 viral vector vaccine^9^). Furthermore, immune response data from PLWH in these trials are currently only available for ChAdOx1^10,11^. Real-world COVID-19 vaccine immune response data from PLWH are also limited. While all three of these vaccines have shown effectiveness following their initial mass rollouts^12–14^, and while clinical trial and observational data have shown robust vaccine-induced humoral immune responses in the general population^15–17^, impaired responses have been reported in certain immunocompromised groups including solid organ transplant recipients^18,19^, cancer patients^20–22^, and individuals on immunosuppressive or immune-depleting therapies^23–25^.

While antiretroviral therapy durably suppresses HIV to undetectable levels in plasma, restores CD4+ T-cell numbers, and can reverse HIV-induced immune dysfunction to a substantial extent^26–29^, persistent immunopathology can nevertheless lead to blunting of immune responses to vaccination in PLWH^30–32^. COVID-19 vaccine immunogenicity data in PLWH are emerging^33–38^, but many of these studies have featured limited numbers of PLWH and/or controls, and few have adjusted for chronic health conditions that may also impair immune responses^39^ (Levy et al.^33^ is an exception). Here, we longitudinally characterize SARS-CoV-2-specific humoral immune responses after COVID-19 immunization in 100 PLWH and 152 control participants ranging from 22 to 88 years of age, with samples analyzed one month after the first and second vaccine doses, and again 3 months after the second dose.

## Results

### Cohort characteristics and COVID-19 vaccine rollout in British Columbia, Canada

Characteristics of the 100 PLWH and 152 controls are shown in Table 1. All PLWH were receiving antiretroviral therapy; the most recent plasma viral load, measured a median of 32 (Interquartile range [IQR] 7–54) days before enrolment, was <50 copies HIV RNA/mL for 95 PLWH, and between 71 and 162 copies/mL for the remaining five PLWH, though prior values were <50 copies/mL in all five of these cases. The most recent CD4+ T-cell count, measured a median of 44 (Interquartile range [IQR] 18–136) days before enrolment, was 710 (IQR 525–935; range 130–1800) cells/mm^3^. The estimated nadir CD4+ T-cell count, recorded a median of 8 (IQR 3.4–15) years before enrolment, was 280 (IQR 120–490; range <10–1010) cells/mm^3^.

**Table 1:** Participant characteristics.

Characteristic	PLWH (*n* = 100)	Controls (*n* = 152)
HIV-related variables		
Receiving antiretroviral therapy, *n* (%)	100 (100%)	–
Most recent plasma viral load, copies HIV RNA/mL, median [IQR]	<50 [<50–<50]	–
Most recent CD4+ T-cell count in cells/mm^3^, median [IQR]	710 [525–935]	–
Nadir CD4+ T-cell count in cells/mm^3^, median [IQR]	280 [120–490]	–
Sociodemographic and health variables		
Age in years, median [IQR]	54 [40–61]	47 [35–70]
Male sex at birth, *n* (%)	88 (88%)	50 (33%)
Ethnicity, n (%)		
White/Caucasian	69 (69%)	78 (51%)
Black	5 (5%)	1 (0.7%)
Asian	10 (10%)	59 (38%)
Latin American	8 (8%)	4 (2.6%)
Middle Eastern/Arab	3 (3%)	0 (0%)
Mixed ethnicity	4 (4%)	8 (5.3%)
Not disclosed	1 (1%)	2 (1.3%)
COVID-19 convalescent (anti-N Ab+) at entry, *n* (%)	8 (8%)	15 (10%)
Number of chronic health conditions, median [IQR]	0 [0–1]	0 [0–1]
Hypertension, *n* (%)	15 (15%)	22 (14.5%)
Diabetes, *n* (%)	6 (6%)	6 (3.9%)
Asthma, *n* (%)	8 (8%)	15 (9.9%)
Obesity, *n* (%)	15 (15%)	14 (9.2%)
Chronic lung disease, *n* (%)	4 (4%)	3 (2%)
Chronic liver disease, *n* (%)	4 (4%)	1 (0.7%)
Chronic kidney disease, *n* (%)	1 (1%)	1 (0.7%)
Chronic heart disease, *n* (%)	1 (1%)	4 (2.6%)
Chronic blood disease, *n* (%)	1 (1%)	2 (1.3%)
Cancer, *n* (%)	5 (5%)	4 (2.6%)
Immunosuppression, *n* (%)	3 (3%)	0 (0%)
At least one of the above, *n* (%)	46 (46%)	50 (33%)
Vaccine-related variables		
mRNA vaccine for first dose, *n* (%)	83 (83%)	148 (97%)
* First dose type*		
BNT162b2, *n* (%)	60 (60%)	133 (87.5%)
mRNA-1273, *n* (%)	23 (23%)	15 (10%)
ChAdOx1, *n* (%)	17 (17%)	4 (2.6%)
mRNA vaccine for second dose, *n* (%)	91 (91%)	151 (99.3%)
Complete vaccine regimen details		
mRNA–mRNA	83 (83%)	148 (97%)
ChAdOx1–mRNA (heterologous)	8 (8%)	3 (2%)
ChAdOx1–ChAdOx1	8 (8%)	1 (0.7%)
ChAdOx1–not disclosed	1 (1%)	–
Time between doses in days, median [IQR]	58 [53–68]	89 [65–98]
Specimen-related variables		
Pre-vaccine specimen, *n* (%)	66 (66%)	148 (97%)
Specimen one month after first dose, *n* (%)	98 (98%)	149 (98%)
Day of collection one month after first dose, median [IQR]	30 [29–32]	30 [28–32]
Specimen one month after second dose, *n* (%)	96 (96%)	151 (99%)
Day of collection one month after second dose, median [IQR]	30 [29–30]	30 [29–32]
Specimen three months after second dose, *n* (%)	92 (92%)	148 (97%)
Day of collection three months after second dose, median [IQR]	90 [90–92]	90 [89–91]

PLWH and controls were similar in terms of age, but were different in terms of sex and ethnicity, with the PLWH group including more males and white ethnicity (Table 1). PLWH and controls had similar burdens of chronic health conditions (median 0; IQR 0–1; range 0–3 conditions in both groups), where 46% and 33% of PLWH and controls, respectively, had at least one condition. The most commonly reported conditions were hypertension, obesity, and asthma. There is some evidence that all three of these conditions can negatively affect immune responses to vaccination against other pathogens^40–44^ as well as COVID-19^45–47^, where high-dose corticosteroid treatments used to treat severe asthma could also blunt vaccine responses^48^. At study entry, 8% of PLWH and 10% of controls were identified as COVID-19 convalescent based on the presence of anti-N antibodies. An additional one (1%) PLWH and four (2.6%) controls developed anti-N antibodies during follow-up consistent with SARS-CoV-2 infection after vaccination. These participants were retained in the “COVID-19 naive at study entry” group, as excluding them did not affect overall results.

All participants received two COVID-19 vaccine doses between December 2020 and August 2021, with 97% of controls receiving an mRNA vaccine for their first dose compared to 83% of PLWH (Table 1). This is because health care workers, who represent 59% of controls, were eligible for vaccination before ChAdOx1 was approved in Canada, while members of the public, including PLWH, received the vaccine(s) recommended for their age group during the mass rollout. More PLWH received heterologous (ChAdOx1/mRNA) regimens compared to controls (8% and 2%, respectively). Heterologous regimens were administered in Canada after mRNA vaccines were universally recommended as second doses^49^ due to reports of rare thrombotic events associated with the ChAdOx1 vaccine^50^. The between-dose interval was also longer for controls (median 89 days, versus 58 for PLWH). This is because the province of British Columbia extended the dose interval to 112 days beginning on March 1, 2021 due to limited vaccine supply^51^. As a result, health care workers who were vaccinated around that time waited the longest for their second doses, while those vaccinated at a later date waited a shorter time between doses, as supply increased. Samples were collected prior to vaccination where possible (66% of PLWH and 97% of controls), one month after the first vaccine dose (98% of both PLWH and controls), one month after the second dose (96% of PLWH and 99% of controls) and again 3 months after the second dose (92% of PLWH and 97% of controls).

### Anti-RBD binding antibody responses after first and second vaccine doses

**Fig. 1:**
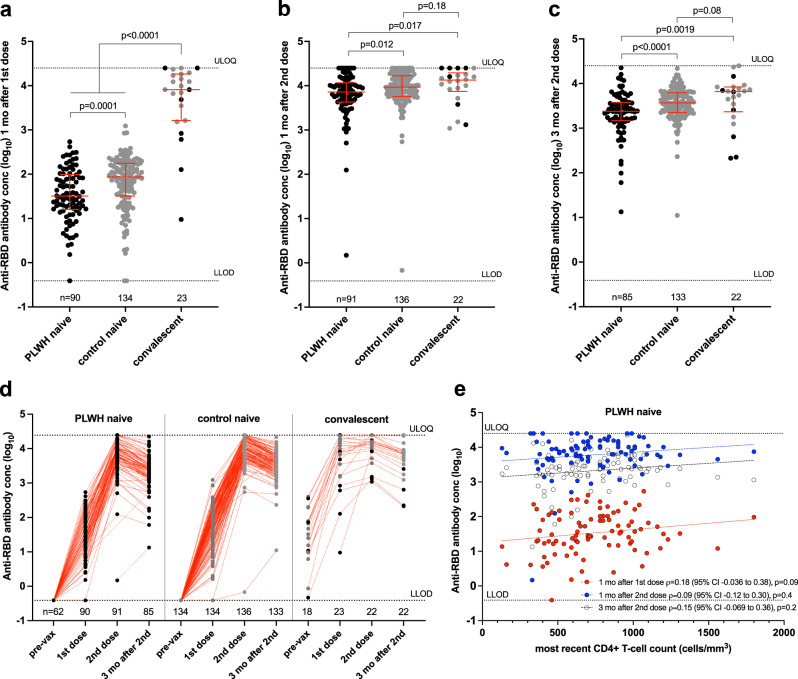
Binding antibody responses to spike RBD following one and two COVID-19 vaccine doses. **a** Binding antibody responses to the SARS-CoV-2 spike RBD in serum one month following the first COVID-19 vaccine dose in PLWH (black circles) and controls (grey circles) who were COVID-19 naive at study entry. Convalescent participants, denoting those with anti-N antibodies at study entry, are presented as a single group but colored by subgroup as above. The numbers of participants analyzed are indicated at the bottom of the plot. Red bars and whiskers represent the median and IQR. *p*-values were computed using the Mann–Whitney *U*-test and are uncorrected for multiple comparisons. *U*-statistics were 4238 (PLWH naive vs. control naive), 93 (PLWH naive vs. convalescent), and 173 (control naive vs. convalescent). LLOD lower limit of detection, ULOQ upper limit of quantification. **b** Binding antibody responses one month after the second dose, presented as in (**a**). *U*-statistics were 4976 (PLWH naive vs. control naive), 675 (PLWH naive vs. convalescent) and 1226 (control naive vs. convalescent). **c** Binding antibody responses three months after the second dose, presented as in (**a**). *U*-statistics were 3841 (PLWH naive vs. control naive), 539 (PLWH naive vs. convalescent) and 1123 (control naive vs. convalescent). **d** Binding antibody responses plotted longitudinally for each group, beginning with the pre-vaccine time point. Red dotted lines connect participants’ longitudinal measurements. The numbers of participants analyzed are indicated at the bottom of the plot. **e** Correlation between most recent CD4+ T-cell count and binding antibody responses one month after the first dose (red circles; *n* = 90), one month after the second dose (blue circles; *n* = 91) and three months following the second dose (clear circles; *n* = 85). Matching-coloured dotted lines help visualize the trend.

Among participants naive to COVID-19 at study entry, all but three (one PLWH and two controls) developed anti-RBD antibodies after one vaccine dose, though overall concentrations in PLWH (median 1.51 [IQR 1.20–1.99] log_10_ U/mL) were on average ~0.4 log_10_ lower than controls (median 1.94 [IQR 1.51–2.25] log_10_ U/mL; Mann–Whitney *p* = 0.0001) (Fig. 1a). In contrast, and consistent with studies demonstrating robust immune responses after one vaccine dose in individuals with prior COVID-19^52,53^, anti-RBD antibody concentrations in COVID-19 convalescent participants (median 3.91 [IQR 3.21–4.26] log_10_ U/mL) were >2 log_10_ higher than in the COVID-19 naive PLWH or control participants (both *p* < 0.0001). In the univariable analyses presented here, convalescent participants were grouped together as there is insufficient power to further stratify by PLWH versus control subgroups, though responses were slightly lower among the former; Mann–Whitney *p* = 0.17). In multivariable analyses controlling for sociodemographic, health and vaccine-related variables, the strongest independent predictors of lower antibody responses after one dose were older age (every decade of age was associated with an adjusted ~0.1 log_10_ lower response; *p* = 0.0002), and a higher number of chronic health conditions (every additional condition associated with an adjusted 0.14 log_10_ lower response; *p* = 0.0053) (Table 2). HIV infection was also associated with an adjusted 0.2 log_10_ lower antibody response after one vaccine dose (*p* = 0.034). Prior COVID-19 was associated with an adjusted 1.88 log_10_ higher response after one dose (*p* < 0.0001).

**Table 2:** Multivariable analyses of the relationship between sociodemographic, health and vaccine-related variables on immunogenicity measures after first and second COVID-19 vaccine doses.

Outcome	Variable	1 mo after 1st: Est (95% CI), *p*^a^	1 mo after 2nd: Est (95% CI), *p*^a^	3 mo after 2nd: Est (95% CI), *p*^a^
Anti-RBD Abs (log_10_)	HIV	−0.20 (−0.38 to −0.015); ***p*** = **0.034**	−0.023 (−0.18 to 0.14); *p* = 0.78	−0.13 (−0.28 to 0.011); *p* = 0.07
	Age (per decade increment)	−0.094 (−0.14 to −0.046); ***p*** = **0.0002**	−0.057 (−0.097 to −0.017); ***p*** = **0.0055**	−0.035 (−0.072 to 0.00097); *p* = 0.056
	Male sex	−0.13 (−0.31 to 0.045); *p* = 0.14	−0.018 (−0.16 to 0.12); *p* = 0.80	0.051 (−0.079 to 0.18); *p* = 0.44
	White ethnicity	−0.12 (−0.28 to 0.037); *p* = 0.13	0.059 (−0.070 to 0.19; *p* = 0.37	0.062 (−0.055 to 0.18); *p* = 0.30
	Chronic cond. (per # incr)	−0.14 (−0.24 to −0.043); ***p*** = **0.0053**	−0.11 (−0.19 to −0.034); ***p*** = **0.0053**	−0.098 (−0.17 to −0.024); ***p*** = **0.01**
	ChAdOx1 as first vaccine	−0.24 (−0.51 to 0.031); *p* = 0.083	–	–
	Dual ChAdOx1 regimen	–	−0.63 (−0.97 to −0.29); ***p*** = **0.0003**	−0.70 (−1.0 to −0.40); ***p*** < **0.0001**
	Dose interval (per week incr)	–	0.025 (0.0022 to 0.047); ***p*** = **0.031**	0.028 (0.0074 to 0.048); ***p*** = **0.008**
	Days since vaccine	0.023 (−0.0011 to 0.047); *p* = 0.061	−0.0022 (−0.024 to 0.020); *p* = 0.84	0.0026 (−0.014 to 0.019); *p* = 0.75
	COVID-19 convalescent	1.88 (1.63 to 2.13); ***p*** < **0.0001**	0.071 (−0.14 to 0.28); *p* = 0.50	0.10 (−0.082 to 0.29); *p* = 0.27
ACE2 displ. (%)^b^	HIV	−10.95 (−20.35 to −1.56); ***p*** = **0.023**	0.64 (−5.274 to 6.547); *p* = 0.83	−6.05 (−16.08 to 3.98); *p* = 0.24
	Age (per decade increment)	−1.47 (−3.14 to 0.41); *p* = 0.13	−1.62 (−2.72 to −0.52); ***p*** = **0.0042**	−2.32 (−4.24 to −0.41); ***p*** = **0.018**
	Male sex	−6.94 (−13.25 to −0.62); ***p*** = **0.031**	−2.17 (−6.09 to 1.77); *p* = 0.28	−0.81 (−7.71 to 6.09); *p* = 0.82
	White Ethnicity	−5.46 (−10.95 to 0.031); *p* = 0.051	1.181 (−2.28 to 4.65); *p* = 0.50	1.50 (−4.51 to 7.51); p = 0.62
	Chronic cond. (per # incr)	−0.85 (−4.29 to 2.58); *p* = 0.63	−2.71 (−4.85 to −0.58); ***p*** = **0.013**	−2.51 (−6.27 to 1.24); *p* = 0.19
	ChAdOx1 as first vaccine	−18.77 (−28.34 to −9.21); ***p*** = **0.0001**	–	–
	Dual ChAdOx1 regimen	–	−29.48 (−38.50 to −20.47); ***p*** < **0.0001**	−33.5 (−48.59 to −18.41); ***p*** < **0.0001**
	Dose interval (per week incr)	–	−0.24 (−0.92 to 0.43); *p* = 0.48	−0.89 (−2.03 to 0.25); *p* = 0.12
	Days since vaccine	0.52 (−0.32 to 1.37); *p* = 0.22	−0.12 (−0.70 to 0.47); *p* = 0.70	−0.41 (−1.28 to 0.45); *p* = 0.35
	EDTA as anticoagulant	6.25 (−3.74 to 16.23); *p* = 0.22	1.17 (−5.57 to 7.90); *p* = 0.73	11.88 (0.50 to 23.25); ***p*** = **0.041**
	COVID-19 convalescent	36.37 (27.68 to 45.05); ***p*** < **0.0001**	2.84 (−2.75 to 8.44); *p* = 0.32	9.35 (−0.048 to 18.76); *p* = 0.051
Viral neut (log_2_)^b,c^	HIV	−0.28 (−0.62 to 0.056); *p* = 0.10	0.17 (−0.51 to 0.84); *p* = 0.63	−0.2 (−0.88 to 0.49); *p* = 0.58
	Age (per decade increment)	−0.047 (−0.11 to 0.017); *p* = 0.15	−0.18 (−0.31 to −0.054); ***p*** = **0.0055**	−0.13 (−0.26 to 0.0043); *p* = 0.058
	Male sex	−0.1 (−0.33 to 0.12); *p* = 0.38	−0.37 (−0.82 to 0.077); *p* = 0.10	0.062 (−0.41 to 0.54); *p* = 0.80
	White ethnicity	0.057 (−0.14 to 0.25); *p* = 0.57	−0.16 (−0.56 to 0.24); *p* = 0.42	−0.032 (−0.45 to 0.38); *p* = 0.88
	Chronic cond. (per # incr)	0.046 (−0.078 to 0.17); *p* = 0.47	−0.29 (−0.54 to −0.047); ***p*** = **0.02**	−0.16 (−0.42 to 0.099); *p* = 0.23
	ChAdOx1 as first vaccine	−0.14 (−0.48 to 0.21); *p* = 0.44	–	–
	Dual ChAdOx1 regimen	–	−1.37 (−2.40 to −0.35); ***p*** = **0.0088**	−1.54 (−2.58 to −0.51); ***p*** = **0.0037**
	Dose interval (per week incr)	–	0.049 (−0.028 to 0.13); *p* = 0.21	−0.0018 (−0.080 to 0.077); *p* = 0.96
	Days since vaccine	0.024 (−0.061 to 0.55); *p* = 0.12	−0.0092 (−0.076 to 0.058); *p* = 0.79	−0.044 (−0.10 to 0.015); *p* = 0.14
	EDTA as anticoagulant	0.3 (−0.061 to 0.66); *p* = 0.1	0.83 (0.061 to 1.60); ***p*** = **0.035**	0.43 (−0.36 to 1.21); *p* = 0.28
	COVID-19 convalescent	3.9 (3.60 to 4.22); ***p*** < **0.0001**	1.07 (0.43 to 1.70); ***p*** = **0.0011**	1.612 (0.97 to 2.26); ***p*** < **0.0001**

^a^For each study visit (one month after the 1st vaccine dose; 1 month after the 2nd dose, and 3 months after the 2nd dose), we report the Estimate (with the 95% Confidence Interval in parentheses); followed by the *p*-value. Statistically significant *p*-values are in bold.

^b^Analyses performed on plasma (i.e. ACE2 displacement and viral neutralization) additionally correct for the anticoagulant used, with ACD as the reference category. Analyses of anti-RBD concentration do not correct for this variable because this assay was performed on serum collected in the same tube type.

^c^For viral neutralization, reciprocal plasma dilutions were log_2_ transformed prior to multivariable analysis.

An extended version of this table, which also lists the *F*-statistics and model degrees of freedom, can be found in the Supplementary information.

The second vaccine dose substantially boosted anti-RBD binding antibody concentrations in all but two participants: one PLWH with immunodeficiency due to a chronic blood disorder, and one >80-year-old control participant with three chronic health conditions (Fig. 1b). One month after the second dose, antibody concentrations in COVID-19 naive PLWH (median 3.86 [IQR 3.63–4.07] log_10_ U/mL) were only ~0.1 log_10_ lower than those in naive controls (median 3.97 [IQR 3.76–4.22] log_10_ U/mL; Mann–Whitney *p* = 0.012), and only ~0.2 log_10_ lower than in convalescent participants (median 4.13 [IQR 3.87–4.29] log_10_ U/mL; Mann–Whitney *p* = 0.017. In multivariable analyses controlling for sociodemographic, health and vaccine-related variables however, HIV infection was no longer associated with antibody concentrations one month after the second vaccine dose (*p* = 0.78, Table 2). Rather, older age, a greater number of chronic conditions and having received two ChAdOx1 doses were independently predictive of weaker responses at this time point, with every 10 years of older age, each additional chronic condition and having received dual ChAdOx1 doses associated with 0.057 log_10,_ 0.11 log_10_ and 0.63 log_10_ lower antibody concentrations, respectively (all *p* < 0.01). A longer dose interval was also associated with marginally higher antibody concentrations at this time point (0.025 log_10_ per additional week, *p* = 0.031). One month after the second dose, there was no longer a significant association between prior COVID-19 and antibody response (*p* = 0.5).

By 3 months following the second vaccine dose, antibody concentrations had declined in all participants by an average of ~0.4–0.5 log_10_ (Fig. 1c, d). Among PLWH and controls who were naive to COVID-19 at study entry, antibody levels had declined to median concentrations of 3.38 [IQR 3.17–3.58] log_10_ U/mL in PLWH and 3.57 [IQR 3.35–3.79] log_10_ U/mL in controls (Mann–Whitney *p* < 0.0001), while those in convalescent participants had declined to a median of 3.82 [IQR 3.37–3.92] log_10_ U/mL (Fig. 1c). The association between HIV infection and lower antibody concentrations at this time point however did not remain statistically significant in multivariable analyses, though the p-value was marginal (*p* = 0.07, Table 2; Supplementary Table 2). Rather, a greater number of chronic conditions and having received two ChAdOx1 doses remained statistically significantly associated with weaker responses at this time point (*p* = 0.01 and *p* < 0.0001, respectively), while a longer dose interval remained associated with higher antibody concentrations (*p* = 0.008).

Among PLWH who were naive to COVID-19 at study entry, we observed a weak positive correlation between the most recent CD4+ T-cell count and antibody concentration after one dose that was not statistically significant (Spearman’s *ρ* = 0.18, *p* = 0.09), but no significant relationship at either time point after the second dose (Fig. 1e). Similarly, we observed a weak positive relationship between *nadir* CD4+ T-cell count and antibody concentration after one dose that was not statistically significant (Spearman’s *ρ* = 0.19, *p* = 0.07), but no significant relationship at either time point after the second dose (Supplementary Fig. 1).

### ACE2 receptor displacement activities after first and second vaccine doses

**Fig. 2:**
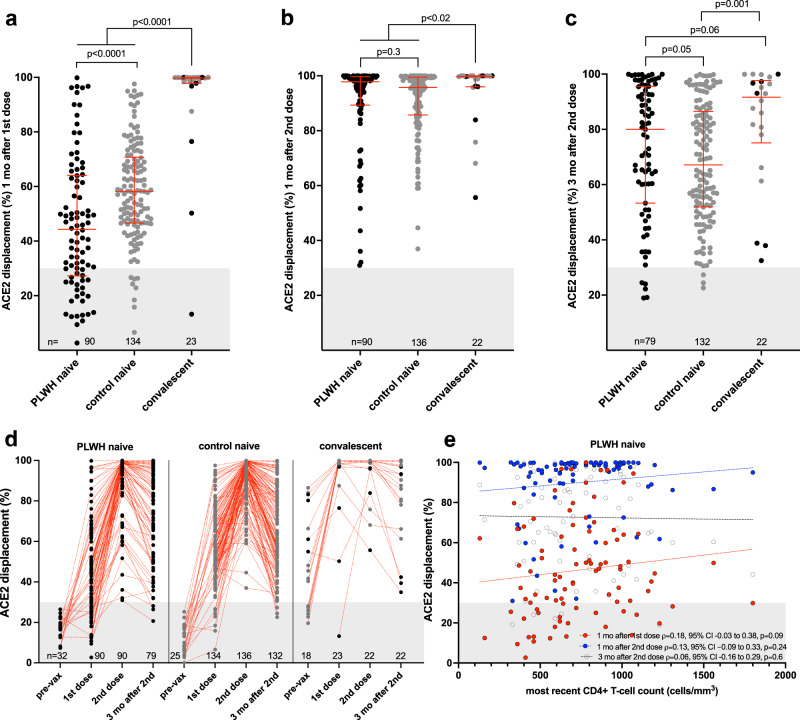
Ability of vaccine-induced antibodies to block ACE2-receptor binding following one and two COVID-19 vaccine doses. **a** ACE2 displacement activities of plasma antibodies one month following the first COVID-19 vaccine dose in PLWH (black circles) and controls (grey circles) who were COVID-19 naive at study entry. Convalescent participants are colored by subgroup. The numbers of participants analyzed are indicated at the bottom of the plot. Red bars and whiskers represent median and IQR. Grey shaded area denotes the range of values observed in pre-vaccine plasma from COVID-19 naive participants (see **d**). *p*-values were computed using the Mann–Whitney *U*-test and are uncorrected for multiple comparisons. *U*-statistics were 4002 (PLWH naive vs. control naive), 144 (PLWH naive vs. convalescent) and 255 (control naive vs. convalescent). **b** ACE2 displacement activities one month after the second dose, presented as in (**a**). *U*-statistics were 5618 (PLWH naive vs. control naive), 660 (PLWH naive vs. convalescent) and 917 (control naive vs. convalescent). **c** ACE2 displacement activities 3 months after the second dose, presented as in (**a**). *U*-statistics were 4353 (PLWH naive vs. control naive), 644 (PLWH naive vs. convalescent) and 839 (control naive vs. convalescent). **d** ACE2 displacement activities plotted longitudinally for each group. Pre-vaccine measurements were performed only on a subset of COVID-19 naive participants to estimate assay background, shown as grey shading. The numbers of participants analyzed are indicated at the bottom of the plot. Red dotted lines connect participants’ longitudinal measurements. **e** Correlation between most recent CD4+ T-cell count and ACE2 displacement activities one month after the first dose (red circles; *n* = 90), one month after the second dose (blue circles; *n* = 90) and three months following the second dose (clear circles; *n* = 79). Matching-coloured dotted lines help visualize the trend.

We next assessed the ability of plasma to block the RBD-ACE2 interaction, which represents a higher throughput approach to estimate potential viral neutralization activity (also referred to as a surrogate viral neutralization test^54^). After one vaccine dose, PLWH and controls who were COVID-19 naive at study entry exhibited median 44% (IQR 27–64%) and 58% (IQR 47–71%) ACE2 displacement activities, respectively, indicating lower function among PLWH (Mann–Whitney *p* < 0.0001) (Fig. 2a). In contrast, convalescent participants exhibited a median 99.7% (IQR 97.8–99.9%) ACE2 displacement activity after one dose (Mann–Whitney *p* < 0.0001 compared to both naive groups). In multivariable analyses, HIV infection remained significantly associated with an adjusted 11% lower ACE2 displacement activity after one vaccine dose (*p* = 0.023), with male sex (adjusted ~7% lower activity compared to female sex, *p* = 0.031) and having received ChAdOx1 as the first dose (adjusted 18.8% lower activity compared to an mRNA vaccine as first dose, *p* = 0.0001) remaining additional independent predictors of lower ACE2 displacement activity. Prior COVID-19 remained associated with an adjusted 36% higher ACE2 displacement activity following one vaccine dose (*p* < 0.0001).

One month following the second vaccine dose, the median ACE2 displacement activity in COVID-19 naive PLWH and controls rose to >95% in both groups and there was no longer a statistically significant difference between them (median 97.8% [IQR 89.3–99.6%] in PLWH versus 95.7% [85.7–99.5%] in controls, Mann–Whitney *p* = 0.3) (Fig. 2b). Furthermore, while the median ACE2 displacement activity in convalescent individuals (median 99.7% [IQR 96.0–100%]) remained statistically significantly higher than both naïve groups (both *p* < 0.02), the magnitude of this difference was marginal. In multivariable analyses, older age, a larger number of chronic conditions, and dual ChAdOx1 vaccination—but not HIV—were independently associated with lower ACE2 displacement function at this time point (adjusted 1.6% lower ACE2 displacement function for every decade of older age, 2.7% lower function for every additional health condition, and 29% lower function for dual ChAdOx1 vaccination; all *p* < 0.02) (Table 2).

By 3 months following the second vaccine dose, ACE2 displacement activity in COVID-19 naive PLWH and controls had declined to medians of 80% [IQR 53–96%] versus 67% [IQR 52–87%] respectively (Mann–Whitney *p* = 0.05), while the activity in convalescent participants had declined to a median of 92% [IQR 75–98%] (Fig. 2c, d). In multivariable analyses correcting for sociodemographic, health and vaccine-related variables, older age and having received two ChAdOx1 doses—but not HIV—were statistically significantly associated with weaker responses at this time point (*p* = 0.018 and *p* < 0.0001, respectively; Table 2).

Among PLWH who were naive to COVID-19 at study entry, we observed a weak positive correlation between recent CD4+ T-cell count and ACE2 displacement activity after one dose that was not statistically significant (Spearman’s *ρ* = 0.18, *p* = 0.09), but no association at either time point after two doses (Fig. 2e). Similarly, we observed a weak positive relationship between *nadir* CD4+ T-cell count and ACE2 displacement activity after one dose that was not statistically significant (Spearman’s *ρ* = 0.2, *p* = 0.06), but no association at either time point following two doses (Supplementary Fig. 1).

### Viral neutralization activity after first and second vaccine doses

**Fig. 3:**
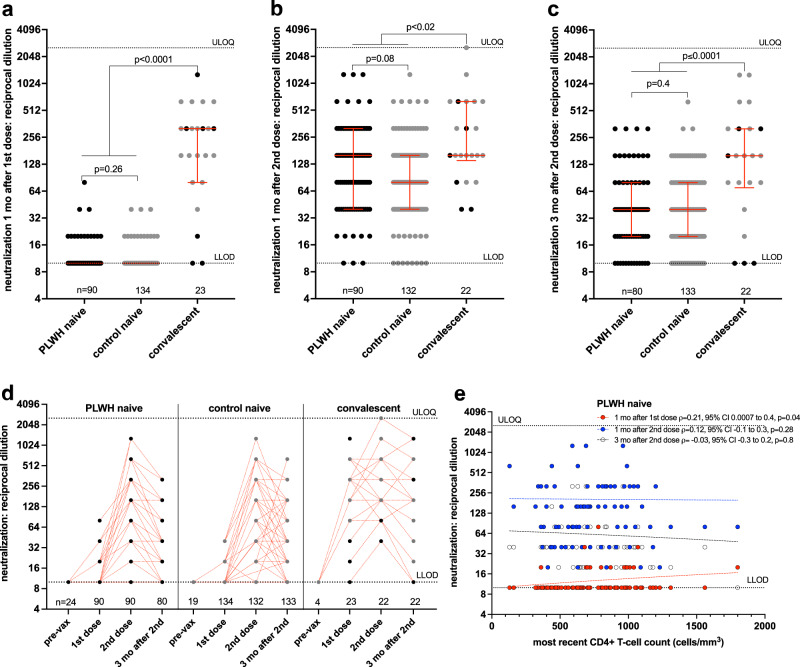
Ability of vaccine-induced antibodies to neutralize live SARS-CoV-2 following one and two COVID-19 vaccine doses. **a** Viral neutralization activities, defined as the highest reciprocal plasma dilution at which neutralization was observed in all triplicate assay wells, one month following the first COVID-19 vaccine dose in PLWH (black circles) and controls (grey circles) who were COVID-19 naive at study entry. Convalescent participants are colored by subgroup. The numbers of participants analyzed are indicated at the bottom of the plot. Red bars and whiskers represent median and IQR. *p*-values were computed using the Mann–Whitney *U*-test and are uncorrected for multiple comparisons. *U*-statistics were 5721 (PLWH naive vs. control naive), 116 (PLWH naive vs. convalescent) and 158 (control naive vs. convalescent). LLOD: assay lower limit of detection. ULOQ: assay upper limit of quantification. **b** Viral neutralization activities one month after the second vaccine dose, presented as in (**a**). U-statistics were 5131 (PLWH naive vs. control naive), 664 (PLWH naive vs. convalescent) and 777 (control naive vs. convalescent). **c** Viral neutralization activities 3 months after the second dose, presented as in (**a**). *U*-statistics were 4984 (PLWH naive vs. control naive), 431 (PLWH naive vs. convalescent) and 686 (control naive vs. convalescent). **d** Viral neutralization activities plotted longitudinally for each group. Pre-vaccine measurements were performed only on a subset of COVID-19 naive and convalescent participants, none of whom had detectable neutralization activity at this time. The numbers of participants analyzed are indicated at the bottom of the plot. Note that many values are superimposed. Red dotted lines connect participants’ longitudinal measurements. **e** Correlation between most recent CD4+ T-cell count and viral neutralization activities one month after the first dose (red circles, *n* = 90), one month after the second dose (blue circles, *n* = 90) and three months following the second dose (clear circles, *n* = 80). Matching-coloured dotted lines help visualize the trend.

After one vaccine dose, plasma from most COVID-19 naive participants displayed weak or no ability to neutralize live SARS-CoV-2, with no significant differences between PLWH and controls (median and IQR undetectable in both groups; Mann–Whitney *p* = 0.26) (Fig. 3a). In contrast, neutralization activities in COVID-19 convalescent individuals were significantly higher, where the median reciprocal plasma dilution needed to achieve neutralization was 320 (IQR 80–320; *p* < 0.0001 compared to both naive groups) (Fig. 3a). Consistent with this, only COVID-19 convalescent status was significantly associated with higher neutralization activity in multivariable analyses after one dose (Table 2).

At one month after the second vaccine dose, viral neutralization activities in COVID-19 naive PLWH and controls increased substantially in both groups, with naive PLWH achieving neutralization at a median reciprocal plasma dilution of 160 (IQR 40–320) compared to a median of 80 (IQR 40–160) in controls (*p* = 0.08) (Fig. 3b). The viral neutralization activities of COVID-19 convalescent individuals (median reciprocal dilution of 160; IQR 140–640) remained marginally higher than COVID-19 naive individuals at this time point (*p* < 0.02 for both comparisons). Consistent with the other humoral functions evaluated, multivariable analyses identified older age, a higher number of chronic conditions, and dual ChAdOx1 vaccination—but not HIV—as being independently associated with lower viral neutralization activity after two COVID-19 vaccine doses (*p* ≤ 0.02; Table 2). COVID-19 convalescent status also remained significantly associated with higher neutralization activity at this time point (*p* = 0.0011).

By 3 months following the second vaccine dose, neutralization activity had declined an average of two- to four-fold in all participants, to median reciprocal dilutions of 40 [IQR 20–80] in both COVID-19 naive PLWH and controls (Mann–Whitney *p* = 0.4), versus 160 [IQR 70–320] in convalescent participants (*p* < 0.0001 for both comparisons) (Fig. 3c, d). In multivariable analyses, the only two variables that were statistically significantly associated with neutralization activity at this time point were having received two ChAdOx1 doses (associated with lower neutralization activity, *p* = 0.0037) and COVID-19 convalescent status (associated with higher neutralization activity, *p* < 0.0001; Table 2).

Among PLWH who were naive to COVID-19 at study entry, we observed a weak positive correlation between recent CD4+ T-cell count and viral neutralization activity after one dose (Spearman’s *ρ* = 0.21, *p* = 0.04), but this association no longer remained at either time point after two doses (Fig. 3e). We observed no significant correlations between nadir CD4+ T-cell count and viral neutralization activity at any time point evaluated (Supplementary Fig. 1).

### Humoral response against the SARS-CoV-2 delta variant

**Fig. 4:**
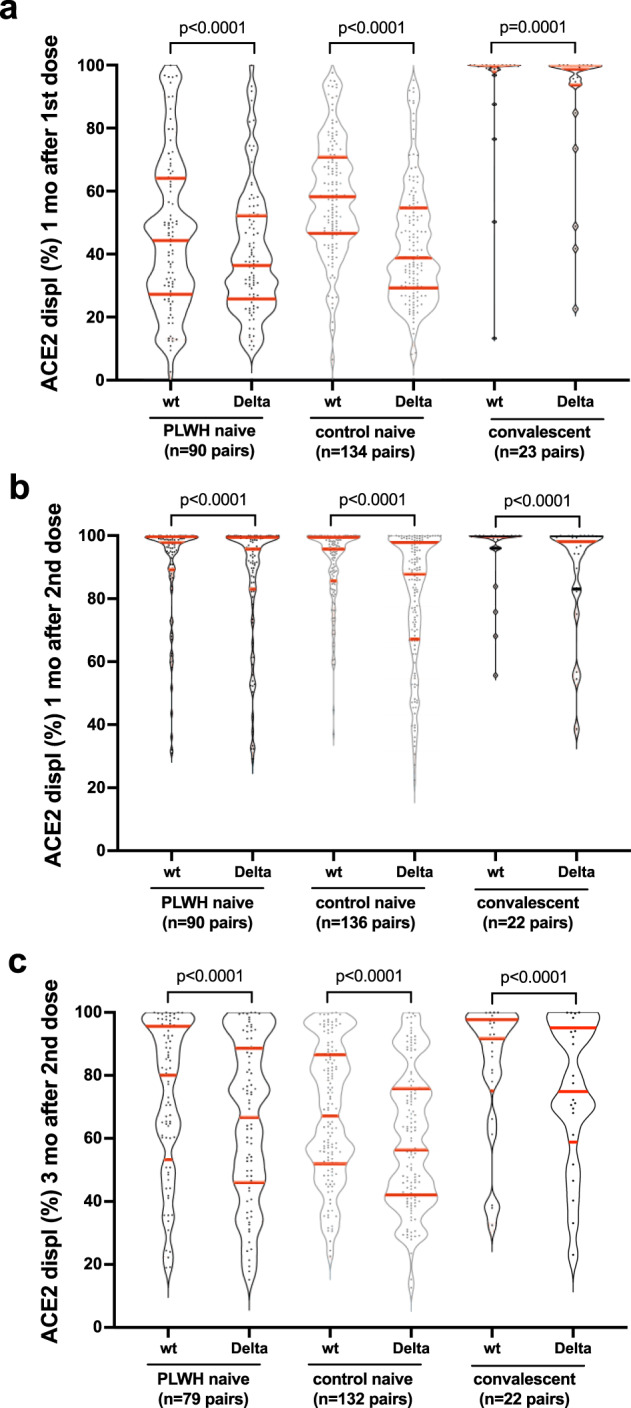
ACE2 displacement activities against the original and Delta SARS-CoV-2 variants after one and two doses of COVID-19 vaccine. **a** ACE2 displacement activities of plasma antibodies against the original wild-type (wt) and Delta variant Spike-RBD in naive PLWH, naive controls, and convalescent individuals one month after the first vaccine dose. Horizontal red lines depict the median, 1st and 3rd quartiles. *p*-values were computed using the Wilcoxon matched-pairs signed rank test and are uncorrected for multiple comparisons. **b** Responses one month after the second vaccine dose, displayed as in (**a**). **c** Responses 3 months after the second vaccine dose, displayed as in (**a**).

Given recent concerns that certain SARS-CoV-2 variants may be more transmissible or evade aspects of host immunity^55,56^, we examined the ACE2 displacement activity in plasma against the widespread B.1.617.2 (Delta) variant. After one vaccine dose, plasma from all groups was impaired in its ability to block ACE2 receptor engagement by the Delta RBD compared to the original wild-type (Wuhan) RBD, where the average (median) magnitude of this impairment was ~5%, ~15% and ~1% for COVID-19 naive PLWH, naive controls and convalescents, respectively (Wilcoxon-matched pairs signed rank test, all *p* ≤ 0.0001) (Fig. 4a). One month after the second vaccine dose, these impairments remained, albeit at a lower magnitude (a median of ~2%, ~7% and ~1% for naive PLWH, naive controls and convalescents, respectively, all *p* < 0.0001; Fig. 4b). By 3 months after the second vaccine dose, the impairments again became more pronounced (a median of ~6%, ~8% and ~6% for naive PLWH, naive controls and convalescents, respectively, all *p* < 0.0001; Fig. 4c). Given the strong correlations between ACE2 displacement and viral neutralization activities observed in our study against the wild-type strain (overall Spearman’s *ρ* = 0.84, *p* < 0.0001; Supplementary Fig. 2), these results suggest that vaccine-elicited humoral responses may be less able to prevent infection by the Delta variant, which is consistent with a recent report showing reduced ability of plasma from convalescent and vaccinated individuals to neutralize this strain^57^.

## Discussion

Our results add to a growing body of evidence that adult PLWH receiving stable antiretroviral therapy, who have suppressed plasma HIV loads and who have CD4+ T-cell counts in a healthy range, generally mount robust initial humoral immune responses to two COVID-19 vaccine doses^10,33–35,37,38^. Though HIV infection was associated with marginally (0.2 log_10_) lower overall anti-RBD antibody concentrations and ~11% lower ACE2 displacement activities following a single vaccine dose after adjustment for sociodemographic, health, and vaccine-related variables, we observed no statistically significant effect of HIV infection on any humoral response at either 1 or 3 months after the second vaccine dose after multivariable adjustment (though at the 3-month time point there was a trend towards lower anti-RBD antibody concentrations in PLWH compared to controls; Table 2). Rather, older age and a higher burden of chronic health conditions were independently associated with weaker humoral responses after two vaccine doses, consistent with previous reports^39,58–61^. Also consistent with previous reports^62,63^, having received two ChAdOx1 doses, as opposed to a heterologous or autologous mRNA vaccine regimen, was associated with significantly lower “peak” humoral responses (measured one month following the second dose). A recent study has reported that, while humoral responses to the mRNA vaccines initially reach high levels but wane considerably thereafter, immune responses induced by a COVID-19 viral vector vaccine induced lower median titers that remained more steady over time^64^, however in our study humoral responses remained significantly lower at 3 months after the second dose in individuals who received two ChAdOx1 vaccines. A longer interval between first and second COVID-19 vaccine doses (where the maximum interval among study participants was 122 days) was also associated with marginally higher binding antibody concentrations, though not ACE2 displacement or viral neutralization activities, which is partially consistent with reports of improved antibody and T-cell responses using extended dosing intervals of the BNT162b2 mRNA vaccine^65^. Indeed, Canada’s unique adoption of a longer (up to 112 days) dose interval yields insight into the magnitude of peak humoral responses following such an extended regimen. It is interesting that the anti-RBD antibody concentrations following two doses measured in the present study are generally higher than in studies of individuals who had shorter dose intervals that also employed the Roche Elecsys Anti-SARS-CoV-2 S assay, even though responses following the first dose were similar^66–69^. Comparing values across studies should be done with caution however, as the assay quantitative range varies based on the maximum sample dilution performed. It is also notable that at 3 months following the second vaccine dose, having COVID-19 prior to vaccination remained strongly associated with higher virus neutralization activity and marginally higher ACE2 displacement activity (Table 2). This is consistent with reports of superior “hybrid immunity” in previously infected individuals after vaccination^70,71^, which is characterized by the presence of higher affinity antibodies that display enhanced neutralization potency and variant cross-recognition activity.

Importantly, among PLWH in our study, all of whom were receiving suppressive antiretroviral treatment, we observed only a very weak positive correlation between the most recent CD4+ T-cell count and humoral responses, and only after the first vaccine dose. This association disappeared following the second vaccine dose. While CD4+ T-cell counts <250 cells/mm^3^ have been associated with lower antibody levels following one COVID-19 vaccine dose^36,72^, and in some studies also after two doses^38^, we were unable to confirm this finding as only two PLWH in the present study had CD4+ T-cell counts in this range, and both mounted strong vaccine responses. Moreover, although we found weak positive correlations between *nadir* CD4+ T-cell counts (which were as low as <10 cells/mm^3^ in our cohort) and both anti-RBD antibody concentrations and ACE2 displacement activities following one dose, these associations no longer remained following the second dose. Furthermore, we observed no association between viral neutralization activity and nadir CD4 T+ cell count after either vaccine dose. Taken together with a recent study that reported a lack of association between nadir CD4+ T-cell count and antibody response after two doses of BNT162b2^38^, this indicates that, for PLWH currently receiving suppressive antiretroviral therapy and whose CD4+ T-cell counts are currently in a healthy range, having had low CD4 T+ cell counts in the past will not necessarily compromise immune responses to COVID-19 vaccines presently.

We also observed that the ability of vaccine-induced plasma antibodies to disrupt the ACE2/RBD interaction was modestly yet significantly reduced against the now widespread SARS-CoV-2 Delta variant compared to the original strain for all participant groups. Given the ability of SARS-CoV-2 variants to evade at least some aspects of vaccine-elicited immunity^55^, this suggests that all individuals, regardless of HIV status, will remain more susceptible to infection by this variant, even after vaccination.

Our study has several limitations. Our results may not be generalizable to PLWH who are not receiving antiretroviral therapy, who have CD4+ T-cell counts <200 cells/mm^3^, or who have complex co-morbidities (54% of PLWH in our study reported no chronic health conditions). Our study did not include children or adolescents living with HIV. As the precise immune correlates of protection for SARS-CoV-2 transmission and disease severity remain incompletely characterized^73^, the implications of our results on individual-level protection from SARS-CoV-2 infection and COVID-19 remain uncertain. The relationship between vaccine-induced antibody concentrations in blood and at mucosal sites, which may be a better correlate of protection, is also incompletely understood, though a recent study identified anti-RBD IgG antibodies in saliva in 100% of participants following a two-dose COVID-19 vaccine series^74^. We did not investigate vaccine-induced T-cell responses, though two recent studies have demonstrated comparable anti-Spike T-cell responses in PLWH compared to controls^10,34^. Our study was not designed nor powered to investigate potential differences in immune responses between the two mRNA vaccines^66,75,76^. Though our study was longitudinal, the latest time point analyzed was 3 months after the second vaccine dose, so additional durability data are needed.

Taken together with existing data^10,33–35^, our results indicate that adults with HIV receiving suppressive antiretroviral therapy and who have CD4+ T-cell counts in the healthy range, mount broadly comparable “peak” humoral immune responses to two COVID-19 vaccine doses compared to individuals without HIV. Furthermore, although immune responses naturally decline over time in all persons, we observed no statistically significant differences between PLWH and controls in the magnitude of any humoral measure at 3 months after the second vaccine dose after multivariable adjustment, though the trend towards marginally lower anti-RBD antibody concentrations (but not other measures) in PLWH at this timepoint underscore the need for ongoing assessments of response durability. Moreover, we found no evidence that a low nadir CD4+ T-cell count negatively influenced the response to COVID-19 vaccination in this group. Rather, our results identified older age, a higher burden of chronic health conditions, and having received a dual ChAdOx1 regimen (as opposed to a heterologous or dual mRNA vaccine regimen)—but not HIV—as significant negative modulators of humoral responses following two-dose COVID-19 vaccination.

These results indicate that PLWH with well-controlled viral loads and CD4+ T-cell counts in a healthy range generally mount strong initial humoral responses to dual COVID-19 vaccination. Factors such as older age, co-morbidities, initial vaccine regimen type, response durability and the rise of new SARS-CoV-2 variants will influence when PLWH (as well as individuals without HIV) will benefit from additional vaccine doses. Further studies of PLWH who are not receiving antiretroviral treatment and/or who have low CD4+ T-cell counts are needed, as are longer-term assessments of vaccine response durability.

## Methods

### Participants and sampling

A total of 100 adult PLWH were recruited through three HIV care clinics in Vancouver, British Columbia (BC), Canada and through community outreach. Control samples were obtained from 152 individuals without HIV, of which 128 were also included in a parallel study of COVID-19 vaccine responses across the adult age spectrum, where the majority (*N* = 89; 59%) were health care workers^77^. HIV-negative status of control participants was determined by self-report. Serum and plasma were collected prior to COVID-19 vaccination (where possible), 1 month after the first COVID-19 vaccine dose, and at 1 and 3 months after the second dose. Plasma was collected in either ethylenediaminetetraacetic acid (EDTA) or anticoagulant citrate dextrose (ACD). Specimens were processed on the day of collection and frozen until analysis. COVID-19 convalescent individuals were identified at study entry by the presence of serum antibodies against the SARS-CoV-2 nucleoprotein (N).

### Ethics approval

This study complied with all relevant ethical regulations for work with human participants, and all participants provided written informed consent. This study was approved by the University of British Columbia/Providence Health Care and Simon Fraser University Research Ethics Boards.

### Data sources

Sociodemographic data, chronic health conditions and COVID-19 vaccination information were collected by self-report and confirmed through medical records where available. We assigned a score of 1 for each of the following 11 chronic health conditions: hypertension, diabetes, asthma, obesity (defined as having a body mass index ≥30), chronic diseases of lung, liver, kidney, heart or blood, cancer, and immunosuppression due to chronic conditions or medication, to generate a total score ranging from 0 to 11. Clinical information for PLWH was recovered from the BC Centre for Excellence in HIV/AIDS Drug Treatment Program Database, which houses clinical records for all PLWH receiving care in BC. For PLWH, having a recent CD4+ T-cell count <200 cells/mm^3^ was classified as “immunosuppression” in the chronic health conditions score.

### Binding antibody assays

We measured total binding antibodies against SARS-CoV-2 N and spike receptor binding domain (RBD) in serum using the Roche Elecsys Anti-SARS-CoV-2 and Elecsys Anti-SARS-CoV-2 S assays, respectively. Both are electro-chemiluminescence sandwich immunoassays. Post-infection, both assays should be positive, whereas post-mRNA vaccination only the S assay should be positive, enabling identification of convalescent samples. The S assay reports results in arbitrary units/mL (U/mL), calibrated against an external standard. For the S assay, the manufacturer indicates that arbitrary unit values can be considered equivalent to international binding antibody units (BAU) as defined by the World Health Organization and the measurement range is from 0.4 to 25,000 U/mL^78^.

### ACE2 displacement assay

We assessed the ability of plasma antibodies to block the interaction between RBD and the ACE2 receptor using the V-plex SARS-CoV-2 Panel 11 (ACE2) kit on a MESO QuickPlex SQ120 instrument (Meso Scale Discovery) at the manufacturer’s recommended 1:20 dilution. ACE2 displacement was calculated as 100−[Arbitrary Units (AU) of ACE2 binding in the presence of plasma/AU of ACE2 binding in the absence of plasma] and reported as a percentage. All samples were assessed in replicate experiments; results from one representative experiment are shown.

### Live virus neutralization assay

Neutralizing activity in plasma was examined using a live SARS-CoV-2 infectivity assay in a Containment Level 3 facility. Assays were performed using isolate USA-WA1/2020 (BEI Resources) and VeroE6-TMPRSS2 (JCRB-1819) target cells. Viral stock was adjusted to 50 TCID_50_/200 µl in DMEM in the presence of serial 2-fold dilutions of plasma (from 1/20 to 1/2560), incubated at 4 °C for 1 h and then added to target cells in 96-well plates in triplicate. Cultures were maintained at 37 °C with 5% CO_2_ and the appearance of viral cytopathic effect (CPE) was recorded 3 days post-infection. Neutralizing activity is reported as the highest reciprocal plasma dilution necessary to prevent CPE in all three replicate wells. Samples exhibiting only partial or no neutralization at the lowest dilution of 1/20 were coded as having a reciprocal dilution of “10”, defined as below the limit of detection in this assay.

### Statistical analysis

Comparisons of binary variables between groups were performed using Fisher’s exact test. Comparisons of continuous variables between groups were performed using the Mann–Whitney *U*-test (for unpaired data) or Wilcoxon test (for paired data). Correlations between continuous variables were performed using Spearman’s correlation. Multiple linear regression was employed to investigate the relationship between sociodemographic, health and vaccine-related variables and humoral outcomes. In addition to HIV infection, variables assessed included age (per decade increment), sex at birth (female as reference group), ethnicity (non-white as reference group), number of chronic health conditions (per number increment), type of vaccine received (mRNA vaccine as reference group), sampling date following vaccine dose (per day increment), and COVID-19 convalescent status at study entry (COVID-19 naive as reference group). Analyses performed following two doses additionally included the interval between doses (per week increment) and having received two ChAdOx1 doses (heterologous or dual mRNA vaccine regimen as the combined reference group). For the assays that tested plasma, which were the ACE2 displacement and viral neutralization assays, the models also corrected for the anticoagulant used (ACD as the reference group). All tests were two-tailed, with *p* < 0.05 considered statistically significant. Analyses were conducted using Prism v9.2.0 (GraphPad).

### Reporting summary

Further information on research design is available in the Nature Research Reporting Summary linked to this article.

## Supplementary information

Supplementary information

REPORTING SUMMARY

## Data Availability

The data supporting the findings of this paper comprise sociodemographic, health, clinical, vaccine and longitudinal laboratory measurement data from participants, including sensitive information such as HIV infection status. To protect participant privacy and confidentiality, data cannot be deposited into the public domain or shared openly. Data can however be shared with interested investigators through existing REB and institutionally approved processes. To request data, please contact the corresponding author with the data request. Data can be shared following completion of REB and institutional requirements (e.g. data transfer agreement). Making datasets of this type available to other researchers through this mechanism is in compliance with our REB and institutional requirements.
