# Supplementary material for: Humoral immune responses to COVID-19 vaccination in people living with HIV receiving suppressive antiretroviral therapy
Source: NPJ Vaccines. 2022 Feb 28;7:28. doi: 10.1038/s41541-022-00452-6 (PMC8885829; doi:10.1038/s41541-022-00452-6)
Supplement: Supplementary file 1 — Supplementary information [file 41541_2022_452_MOESM1_ESM.pdf]

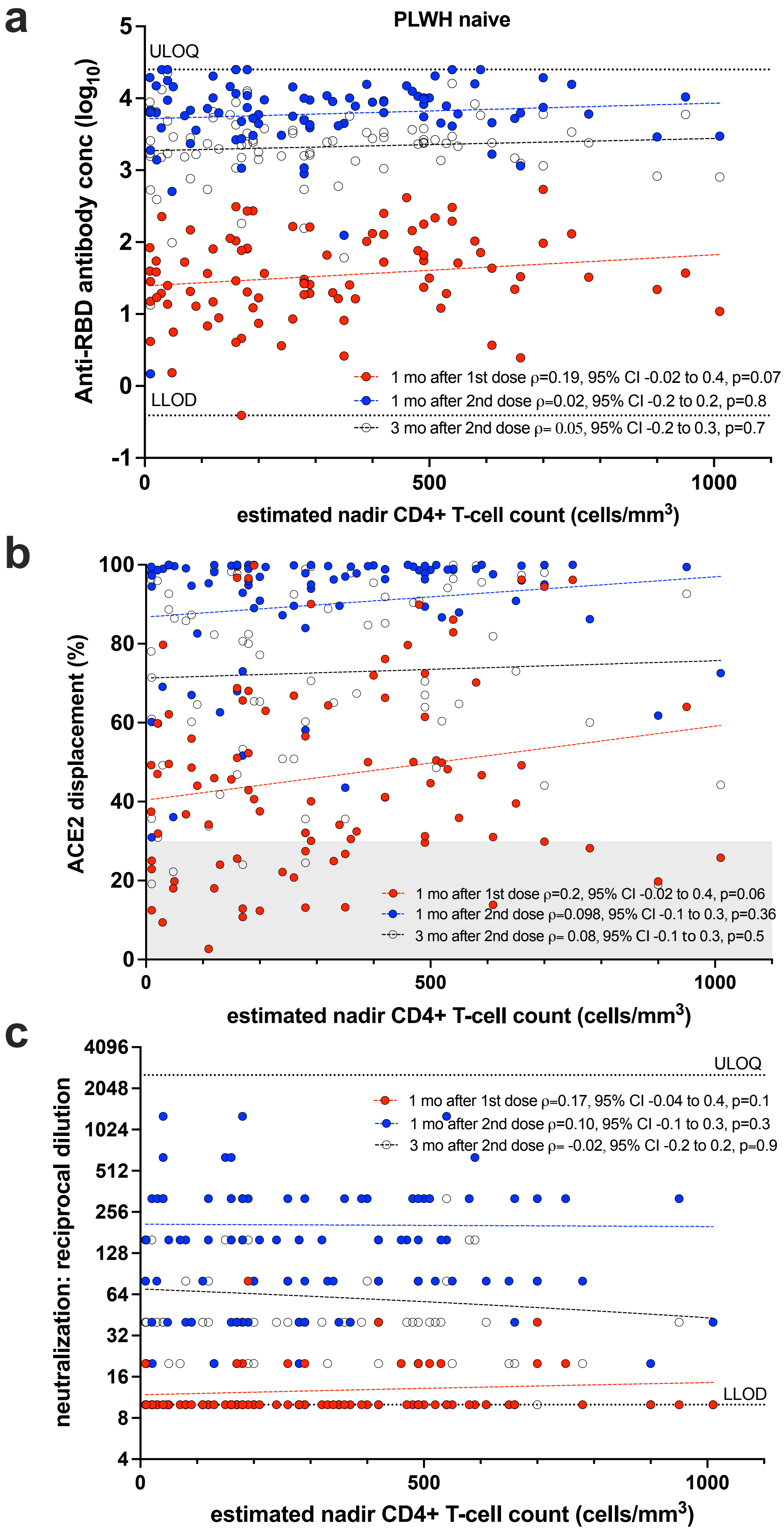

Supplemental Figure 1: Correlation between nadir CD4+ T-cell count and COVID-19 vaccine responses. *Panel A:* Correlation between nadir CD4+ T-cell count and binding antibody responses one month after the first vaccine dose (red circles), one month after the second dose (blue circles) and three months following the second dose (clear circles). Matching-coloured dotted lines help visualize the trend. LLOD: lower limit of detection. ULOQ: upper limit of quantification. *Panel B*: same as A, but showing ACE2 displacement activity. Grey shading denotes assay background levels determined by testing pre-vaccine samples from COVID-19 naive individuals. *Panel C*: same as A, but showing viral neutralization activity.

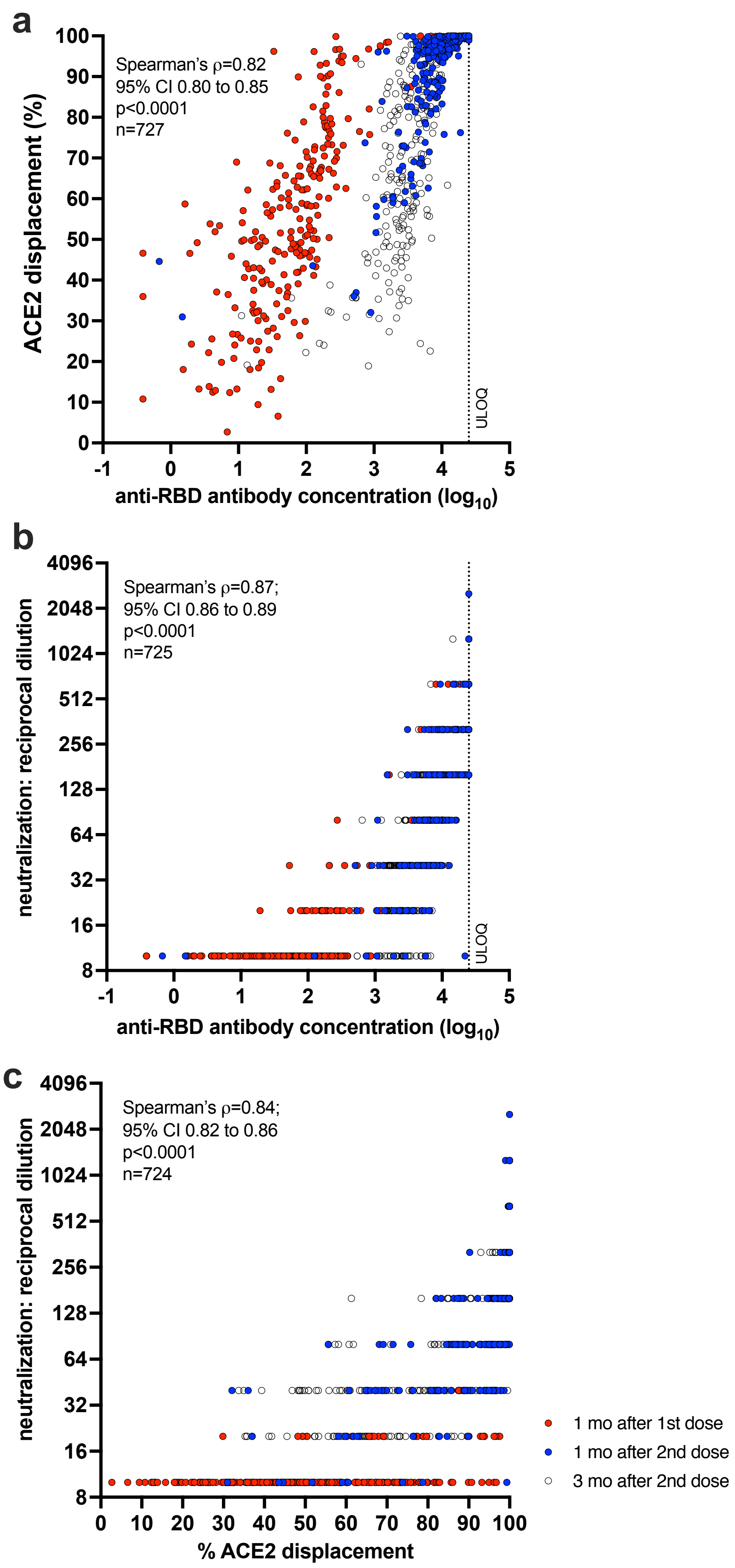

Supplemental Figure 2: Correlations between humoral measures after one and two doses of COVID-19 vaccine

Supplementary (Extended) Table 2: Multivariable analyses of the relationship between sociodemographic, health and vaccine-related variables on immunogenicity measures after first and second COVID-19 doses

Humoral measure	Variable	Time point											
		1 month after 1st dose				1 month after 2nd dose				3 months after 2nd dose			
		F[1]	Estimate	95% CI	p-value	F[1]	Estimate	95% CI	p-value	F[1]	Estimate	95% CI	p-value
**anti-RBD Abs (log10)**	HIV	4.6	-0.20	-0.38 to -0.015	**0.034**	0.08	-0.023	-0.18 to 0.14	0.78	3.3	-0.13	-0.28 to 0.011	0.07
	Age (per decade increment)	15	-0.094	-0.14 to -0.046	**0.0002**	7.8	-0.057	-0.097 to -0.017	**0.0055**	3.7	-0.035	-0.072 to 0.00097	0.056
	Male sex	2.2	-0.13	-0.31 to 0.045	0.14	0.06	-0.018	-0.16 to 0.12	0.80	0.6	0.051	-0.079 to 0.18	0.44
	White Ethnicity	2.3	-0.12	-0.28 to 0.037	0.13	0.8	0.059	-0.070 to 0.19	0.37	1.1	0.062	-0.055 to 0.18	0.3
	Chronic cond. (per # incr)	7.9	-0.14	-0.24 to -0.043	**0.0053**	7.9	-0.11	-0.19 to -0.034	**0.0053**	6.7	-0.098	-0.17 to -0.024	**0.01**
	ChAdOx1 as first vaccine	3.0	-0.24	-0.51 to 0.031	0.083	-	-	-	-	-	-	-	-
	Dual ChAdOx1 regimen	-	-	-	-	13	-0.63	-0.97 to -0.29	**0.0003**	21	-0.70	-1.0 to -0.40	**<0.0001**
	Dose interval (per week incr)	-	-	-	-	4.7	0.025	0.0022 to 0.047	**0.031**	7.3	0.028	0.0074 to 0.048	**0.008**
	Days since vaccine	3.5	0.023	-0.0011 to 0.047	0.061	0.04	-0.0022	-0.024 to 0.020	0.84	0.1	0.0026	-0.014 to 0.019	0.75
	COVID-19 convalescent	219	1.88	1.63 to 2.13	**<0.0001**	0.5	0.071	-0.14 to 0.28	0.50	1.2	0.10	-0.082 to 0.29	0.27
**ACE2 displ. (%)[2]**	HIV	5.3	-10.95	-20.35 to -1.56	**0.023**	0.05	0.64	-5.274 to 6.547	0.83	1.4	-6.05	-16.08 to 3.98	0.24
	Age (per decade increment)	2.3	-1.47	-3.14 to 0.41	0.13	8.4	-1.62	-2.72 to -0.52	**0.0042**	5.7	-2.32	-4.24 to -0.41	**0.018**
	Male sex	4.7	-6.94	-13.25 to -0.62	**0.031**	1.2	-2.17	-6.09 to 1.77	0.28	0.05	-0.81	-7.71 to 6.09	0.82
	White Ethnicity	3.8	-5.46	-10.95 to 0.031	0.051	0.5	1.181	-2.28 to 4.65	0.50	0.2	1.50	-4.51 to 7.51	0.62
	Chronic cond. (per # incr)	0.2	-0.85	-4.29 to 2.58	0.63	6.3	-2.71	-4.85 to -0.58	**0.013**	1.7	-2.51	-6.27 to 1.24	0.19
	ChAdOx1 as first vaccine	15	-18.77	-28.34 to -9.21	**0.0001**	-	-	-	-	-	-	-	-
	Dual ChAdOx1 regimen	-	-	-	-	42	-29.48	-38.50 to -20.47	**<0.0001**	19	-33.5	-48.59 to -18.41	**<0.0001**
	Dose interval (per week incr)	-	-	-	-	0.5	-0.24	-0.92 to 0.43	0.48	2.4	-0.89	-2.03 to 0.25	0.12
	Days since vaccine	1.5	0.52	-0.32 to 1.37	0.22	0.2	-0.12	-0.70 to 0.47	0.70	0.9	-0.41	-1.28 to 0.45	0.35
	EDTA as anticoagulant	1.5	6.25	-3.74 to 16.23	0.22	0.1	1.17	-5.57 to 7.90	0.73	4.2	11.88	0.50 to 23.25	**0.041**
	COVID-19 convalescent	68	36.37	27.68 to 45.05	**<0.0001**	1.0	2.84	-2.75 to 8.44	0.32	3.8	9.35	-0.048 to 18.76	0.051
**Viral neut. (log2)[2,3]**	HIV	2.7	-0.28	-0.62 to 0.056	0.10	0.2	0.17	-0.51 to 0.84	0.63	0.3	-0.2	-0.88 to 0.49	0.58
	Age (per decade increment)	2.1	-0.047	-0.11 to 0.017	0.15	7.8	-0.18	-0.31 to -0.054	**0.0055**	3.6	-0.13	-0.26 to 0.0043	0.058
	Male sex	0.8	-0.1	-0.33 to 0.12	0.38	2.7	-0.37	-0.82 to 0.077	0.10	0.07	0.062	-0.41 to 0.54	0.80
	White Ethnicity	0.3	0.057	-0.14 to 0.25	0.57	0.6	-0.16	-0.56 to 0.24	0.42	0.02	-0.032	-0.45 to 0.38	0.88
	Chronic cond. (per # incr)	0.5	0.046	-0.078 to 0.17	0.47	5.5	-0.29	-0.54 to -0.047	**0.02**	1.5	-0.16	-0.42 to 0.099	0.23
	ChAdOx1 as first vaccine	0.6	-0.14	-0.48 to 0.21	0.44	-	-	-	-	-	-	-	-
	Dual ChAdOx1 regimen	-	-	-	-	7.0	-1.37	-2.40 to -0.35	**0.0088**	8.6	-1.54	-2.58 to -0.51	**0.0037**
	Dose interval (per week incr)	-	-	-	-	1.6	0.049	-0.028 to 0.13	0.21	0.002	-0.0018	-0.080 to 0.077	0.96
	Days since vaccine	2.5	0.024	-0.061 to 0.55	0.12	0.07	-0.0092	-0.076 to 0.058	0.79	2.2	-0.044	-0.10 to 0.015	0.14
	EDTA as anticoagulant	2.7	0.3	-0.061 to 0.66	0.1	4.5	0.83	0.061 to 1.60	**0.035**	1.2	0.43	-0.36 to 1.21	0.28
	COVID-19 convalescent	607	3.9	3.60 to 4.22	**<0.0001**	11	1.07	0.43 to 1.70	**0.0011**	24	1.612	0.97 to 2.26	**<0.0001**

[1] This table contains the same information as Table 2 in the main manuscript, but in compliance with journal reporting requirements, it additionally includes the F statistic (F) for each variable and Model Degrees of Freedom. Model Degrees of freedom (DF) were: Anti-RBD Abs: 238 (1 mo after 1st dose), 239 (1 mo after 2nd dose), 230 (3 mo after 2nd dose); ACE2 displacement: 237 (1 mo after 1st dose), 237 (1 mo after 2nd dose), 222 (3 mo after 2nd dose); Viral neutralization: 237 (1 mo after 1st dose), 233 (1 mo after 2nd dose), 224 (3 mo after 2nd dose). Statistically significant p-values are in bold.

[2]analyses performed on plasma (*i.e.* ACE2 displacement and viral neutralization) also correct for the anticoagulant used, with ACD as the reference category. Analyses of anti-RBD concentration do not correct for this variable because this assay was performed on serum collected in the same tube type.

[3]for viral neutralization, reciprocal plasma dilutions were $\log_2$ transformed prior to multivariable analysis.
