# Supplementary material for: Humoral immune responses to COVID-19 vaccination in people living with HIV receiving suppressive antiretroviral therapy
Source: NPJ Vaccines. 2022 Feb 28;7:28. doi: 10.1038/s41541-022-00452-6 (PMC8885829; doi:10.1038/s41541-022-00452-6)
Supplement: Supplementary file 2 — REPORTING SUMMARY [file 41541_2022_452_MOESM2_ESM.pdf]

nature portfolio

Corresponding author(s): Zabrina Brumme

Last updated by author(s): Jan 10, 2022

Reporting Summary

Nature Portfolio wishes to improve the reproducibility of the work that we publish. This form provides structure for consistency and transparency in reporting. For further information on Nature Portfolio policies, see our Editorial Policies and the Editorial Policy Checklist.

Statistics

For all statistical analyses, confirm that the following items are present in the figure legend, table legend, main text, or Methods section.

n/a	Confirmed	
☐	☒	The exact sample size (*n*) for each experimental group/condition, given as a discrete number and unit of measurement
☐	☒	A statement on whether measurements were taken from distinct samples or whether the same sample was measured repeatedly
☐	☒	The statistical test(s) used AND whether they are one- or two-sided<br>*Only common tests should be described solely by name; describe more complex techniques in the Methods section.*
☐	☒	A description of all covariates tested
☐	☒	A description of any assumptions or corrections, such as tests of normality and adjustment for multiple comparisons
☐	☒	A full description of the statistical parameters including central tendency (e.g. means) or other basic estimates (e.g. regression coefficient) AND variation (e.g. standard deviation) or associated estimates of uncertainty (e.g. confidence intervals)
☐	☒	For null hypothesis testing, the test statistic (e.g. *F*, *t*, *r*) with confidence intervals, effect sizes, degrees of freedom and *P* value noted<br>*Give P values as exact values whenever suitable.*
☒	☐	For Bayesian analysis, information on the choice of priors and Markov chain Monte Carlo settings
☒	☐	For hierarchical and complex designs, identification of the appropriate level for tests and full reporting of outcomes
☐	☒	Estimates of effect sizes (e.g. Cohen's *d*, Pearson's *r*), indicating how they were calculated

Our web collection on statistics for biologists contains articles on many of the points above.

Software and code

Policy information about availability of computer code

Data collection	Binding antibody data (Roche Elecsys) was collected using cobas6000 software version 05-02. ACE2 displacement data (MesoScale Diagnostics) was collected using Methodical Mind Reader SQ120, version 1.0.36. Viral neutralization data (binary scoring of cell cultures for viral cytopathic effects) were recorded using Microsoft OneNote and Excel (Microsoft 365). No custom software was used for data collection.
Data analysis	ACE2 displacement data were analyzed using Discovery Workbench 4.0.13. Analysis of study data was conducted using Prism v9.2.0 (GraphPad). No custom software was used for data analysis.

For manuscripts utilizing custom algorithms or software that are central to the research but not yet described in published literature, software must be made available to editors and reviewers. We strongly encourage code deposition in a community repository (e.g. GitHub). See the Nature Portfolio guidelines for submitting code & software for further information.

Data

Policy information about availability of data

All manuscripts must include a data availability statement. This statement should provide the following information, where applicable:

- Accession codes, unique identifiers, or web links for publicly available datasets
- A description of any restrictions on data availability
- For clinical datasets or third party data, please ensure that the statement adheres to our policy

All data generated in this study are available from the corresponding author upon reasonable request and in accordance with REB and institutional data sharing requirements. Also, as per funding requirements, upon completion of the full study (March 31, 2023), study data will be deposited in a national database administered by the COVID-19 Immunity Task Force.

Field-specific reporting

Please select the one below that is the best fit for your research. If you are not sure, read the appropriate sections before making your selection.

☒ Life sciences ☐ Behavioural & social sciences ☐ Ecological, evolutionary & environmental sciences

For a reference copy of the document with all sections, see nature.com/documents/nr-reporting-summary-flat.pdf

Life sciences study design

All studies must disclose on these points even when the disclosure is negative.

Sample size	An initial power estimate was performed on a recruitment target of N=200 (100 PLWH, 100 controls) based on IgG anti-Spike RBD responses measured via an in house Luminex ELISA-based assay following one dose of BNT162b2, assuming a mean 3.56log10 MFI (arbitrary units) in controls following one dose, with a SD of 0.38 log10. With a sample size of 200 we estimated we would have 80% power to detect as low as a 0.15 log10 difference in binding Ab responses between participants with and without HIV at alpha=0.05. We ultimately exceeded this recruitment target (100 PLWH and 152 controls).
Data exclusions	No data were excluded from analysis. Ns of samples analyzed differ slightly between humoral measures in some cases because samples were aliquoted in batches for analysis, such that samples collected following the aliquot cut-off date were not included in that batch
Replication	Where possible, we replicated some of the data using alternative methods, either commercial or in-house assays. Namely, to verify binding antibody data collected using the Roche Elecsys anti-S assay, we additionally quantified plasma IgG binding antibodies against RBD using the Meso Scale Diagnostics V-plex SARS-CoV-2 (IgG) ELISA assay on a subset of samples and confirmed strong correlations. To verify ACE2 displacement data collected using the Meso Scale Diagnostics system, we additionally assessed this parameter using an in-house Luminex-based assay and confirmed strong correlations. Finally, and as expected, we observed strong correlations between binding antibodies, ACE2 displacement activity and viral neutralization activity in the study samples; these data are presented in Supplemental Figure 2.
Randomization	As this was an observational study, not a clinical trial, participants were not randomized into groups. Covariates were controlled for in multivariable analyses, which are described in detail in the methods with results presented in Table 2.
Blinding	As this was an observational study, not a clinical trial, investigators were not blinded as to the HIV status of study participants during data analysis. As participants were recruited consecutively, and assigned consecutive IDs that did not explicitly incorporate information on their HIV status, prior COVID-19 infection or vaccination regimen, laboratory technicians were effectively blinded as to participant characteristics during data collection.

Reporting for specific materials, systems and methods

We require information from authors about some types of materials, experimental systems and methods used in many studies. Here, indicate whether each material, system or method listed is relevant to your study. If you are not sure if a list item applies to your research, read the appropriate section before selecting a response.

Materials & experimental systems

n/a	Involved in the study	
☒	☐	Antibodies
☐	☒	Eukaryotic cell lines
☒	☐	Palaeontology and archaeology
☒	☐	Animals and other organisms
☐	☒	Human research participants
☒	☐	Clinical data
☒	☐	Dual use research of concern

Methods

n/a	Involved in the study	
☒	☐	ChIP-seq
☒	☐	Flow cytometry
☒	☐	MRI-based neuroimaging

Eukaryotic cell lines

Policy information about cell lines

Cell line source(s)	The VeroE6-TMPRSS2 (JCRB-1819) cells used in the neutralization assay were obtained from the Japanese National Institutes of Biomedical innovation, health and nutrition JCRB cell bank in April 2020.
Authentication	Cell lines distributed by the JCRB are comprehensively qualified by testing microbial contamination, virus contamination and cross culture contamination. Some cells are also characterized by karyotyping and/or cell surface markers. Information is available on the JRCB website at (https://cellbank.nibiohn.go.jp/english/about_e/). No further in-house authentication was performed following receipt of the cells.
Mycoplasma contamination	Cell line was certified negative for mycoplasma contamination by the JCRB at time of shipment to our laboratory in April

Mycoplasma contamination	2020.
Commonly misidentified lines<br>(See ICLAC register)	The VeroE6-TMPRSS2 (JCRB-1819) cell line is not on the list of commonly misidentified lines in the ICLAC register

Human research participants

Policy information about studies involving human research participants

Population characteristics	The covariate-relevant characteristics of the study population are detailed in Table 1 of the manuscript as well as the methods.
Recruitment	Participants living with HIV were recruited via advertisements in HIV clinics in the Metro Vancouver area, through email communications to clinic and community mail-lists, and by outreach to community organizations. Participants without HIV were recruited through electronic communications disseminated through institutional channels and advertisements placed in the community. The method of recruiting participants with HIV could have biased recruitment to individuals receiving care; indeed all participants with HIV were receiving antiretroviral treatment and the majority had CD4 counts in a healthy range. This is explicitly noted in the study, which emphasizes that further study of people with HIV who are not receiving therapy and/or who have low CD4 T-cell counts are needed.
Ethics oversight	This study was approved by the University of British Columbia/Providence Health Care and Simon Fraser University Research Ethics Boards

Note that full information on the approval of the study protocol must also be provided in the manuscript.
